# Steganographic Analysis of Blockchains †

**DOI:** 10.3390/s21124078

**Published:** 2021-06-13

**Authors:** Alexandre Augusto Giron, Jean Everson Martina, Ricardo Custódio

**Affiliations:** 1 Department of Computer Science, Federal University of Technology–Parana (UTFPR), 85902-490 Toledo, PR, Brazil; 2 Graduate Program on Computer Science, Department of Informatics and Statistics, Federal University of Santa Catarina (UFSC), 88040-370 Florianópolis, SC, Brazil; jean.martina@ufsc.br (J.E.M.); ricardo.custodio@ufsc.br (R.C.)

**Keywords:** blockchain, steganography, steganalysis, bitcoin, ethereum

## Abstract

Steganography is one of the ways to hide data between parties. Its use can be worrisome, e.g., to hide illegal communications. Researchers found that public blockchains can be an attractive place to hide communications; however, there is not much evidence of actual use in blockchains. Besides, previous work showed a lack of steganalysis methods for blockchains. In this context, we present a steganalysis approach for blockchains, evaluating it in Bitcoin and Ethereum, both popular cryptocurrencies. The main objective is to answer if one can find steganography in real case scenarios, focusing on LSB of addresses and nonces. Our sequential analysis included 253 GiB and 107 GiB of bitcoin and ethereum, respectively. We also analyzed up to 98 million bitcoin clusters. We found that bitcoin clusters could carry up to 360 KiB of hidden data if used for such a purpose. We have not found any concrete evidence of hidden data in the blockchains. The sequential analysis may not capture the perspective of the users of the blockchain network. In this case, we recommend clustering analysis, but it depends on the clustering method’s accuracy. Steganalysis is an essential aspect of blockchain security.

## 1. Introduction

The use of blockchains in the infrastructure of online services is currently trending. Financial services were the kick start after 2008, propelled by the Bitcoin cryptocurrency [1], but other services are beginning to explore the benefits of blockchains. It seems that society had a demand for strong integrity and decentralized availability of data, where the blockchain fits today as a promising solution. Applications include smart contracts, proof of existing services, supply chains, Peer-to-Peer (P2P) energy trading, house rental, and even secret communications [2,3,4,5,6,7,8,9,10]. One of the arguments favoring blockchains is to give applications increased security and independence on third parties.

Blockchain is a decentralized digital registry with tamper protection. Blocks of data are linked together in a chain, where the valid chain is the one that the majority of participants have an agreement [11]. It combines linking the data with cryptography services, and this combination provides a robust infrastructure for applications. Two of the main benefits of incorporating a blockchain are a guarantee that one cannot change the data once registered and third parties’ independence for this purpose.

Several attacks on cryptocurrencies and blockchains are present in the literature that threaten their security [12]. One of the possible attacks is that the blockchains are subject to entering questionable data. For instance, the work of Matzutt et al. [13] showed the insertion of at least 1600 arbitrary files inside of the Bitcoin blockchain. The authors argued that some of these files contain links to objectionable content. Smart-contracts of the Ethereum cryptocurrency, as another example, require storage for the program data. Sato et al. [14] discuss the “poisoning attack” problem: inserting malicious programs or data in the Ethereum blockchain. Such insertion problems become particularly worrying since the data is made available to all of the network participants. The blockchains’ cost regarding strong integrity is the computational infeasibility of removing data after a poisoning incident. This threat is of particular concern when using public blockchains.

In this context, the detection and prevention of such incidents are paramount. Countermeasures exist and include content filters, editable blockchains, self-verifying account identifiers, and increased transaction rates [15]. Some countermeasures can be implemented and executed by the blockchain miners, which can filter content, check blacklists that can ban malicious participants, and redactable designs which would allow deleting blockchain data [16,17].

The problem of inappropriate data insertion becomes even more worrying if such methods combine with data hiding techniques. Instead of a “plain” insertion, one can use steganography, a set of techniques to hide that communication is taking place. Researchers have proposed approaches for steganography in blockchains [5,18]. By exploiting the blockchains as steganographic channels, such approaches camouflage the communicated data. If not detected a priori, the data can be stored definitely in the blockchain. Therefore, steganography increases the difficulty of the arbitrary data insertion problem in blockchains.

The first approaches for steganography in blockchains appeared almost ten years after the release of the first blockchain. Examples are the Partala’s approach [5] and the ChainChannels of Frkat et al. [19], both in 2018. Not only theoretical constructions but real cases also exist. For instance, members of WikiLeaks used Bitcoin to hide a message, spread in five addresses of a Bitcoin transaction: “1WeRe 1Fine 18chaN 1PoSt 1FAke” [20]. Allegedly, there are legit purposes for steganography in blockchains. The advantages of using blockchains as a steganographic channel, compared to other cover mediums, are listed below [5]:

- Free access with a degree of anonymity offered by, in particular, public blockchains. Besides, many blockchains are being in use today, thus increasing the available networks that steganography users could explore.
- Independence of third parties: in theory, no central authority can control the entirety of the blockchain due to its decentralized design.
- After the participants agreed to publish a block in the chain, no one can delete the data. Therefore, this feature can be attractive for political activists and protesters. They could communicate secretly with resistance against censorship of authoritarian regimes.

Going back to the problem stated by Matzutt et al. [13], where arbitrary data insertion can be problematic, hiding data steganographically in blockchains can be viewed as a similar problem but with additional risks. First, the hiding technique used may overcome the countermeasures against data insertion. From an attacker’s perspective, one can insert inappropriate data to damage the blockchain’s reputation, or to make a cryptocurrency loose its value. Although the attacker does not need steganography for this, he could use it to try to bypass potential content filters. Secondly, public blockchains make the data accessible by any participant of the network, making it easier to spread the hidden information. Thirdly, the disclosure of hidden data may not be easy to perform without a proper detection mechanism.

The term *steganalysis* refers to the mechanisms designed to detect steganography in a communication channel [21]. It gives evidence of steganography use, generally by exploring statistical properties of the communication channel. If the steganographic technique incurs changes in the channel structure, we may observe these changes in the analysis. When observed, the analysis then defeats the primary goal of steganography because it cannot hide the communication anymore. The second step of steganalysis is to reverse the steganography to extract information from the observed changes; if successful, it discloses the secret communication.

### 1.1. Summary of Previous Work

We searched the Bitcoin cryptocurrency for evidence of steganography use. We divided part of the blockchain (253.38 GiB of blocks) into data chunks. The amount of data present in blockchains shows a first difficulty to detect steganography, and then a tool was developed to automate the analysis. Each chunk was analyzed statistically and forensically, but the experiments have found no evidence of steganography.

We defined the analysis scope in previous work by two experiments: checking LSB of Addresses and Checking the Nonces. Both experiments were analyzed statistically, followed by the Scalpel tool [22] to find pieces of evidence of manipulation of these bits to store hidden data. In the first experiment, the LSB of addresses was extracted and analyzed. In the second experiment, we analyzed the Nonce field of Bitcoin blocks statistically, similar to the analysis published in bitslog.com [23]. Standard steganography (such as in images and videos) was not in the scope of the analysis. In both experiments, the analysis did not return concrete evidence of steganography use in Bitcoin.

One of the lessons learned from the previous work is the lack of a steganalysis approach specific to blockchains. Due to this gap, the previous work’s steganalysis was based only on a simple statistical analysis. To the best of our knowledge, no steganalysis approach specific for blockchains has appeared in the literature. This gap means that blockchain networks are currently unassisted of detection mechanisms. The steganographic analysis is essential, for instance, to detect inappropriate content from being communicated (and stored) secretly in the blockchain.

### 1.2. Contributions

This paper aims to fill the gap found in previous work: we propose an approach for steganographic analysis (a.k.a. steganalysis) in blockchains. First, we extend the search for steganographic evidence in Bitcoin but now including blockchain clustering techniques in the analysis. The main focus is to find evidence of steganography use (if there is any) using the proposed approach. Secondly, we generalize the approach, including a second case study, where the Ethereum cryptocurrency was the analysis subject. We also include a forensic analysis for completeness, turning it capable of retrieving content hidden with steganography. Lastly, we discuss the evaluation of the approach. In summary, the main contributions of this paper are:

- classification of the available techniques for data hiding in blockchains;
- a steganalysis approach which considers specific aspects of blockchains;
- a discussion about the limitations, performance constraints, and effectiveness of the proposed approach;
- a search for steganographic evidence in the Bitcoin and the Ethereum blockchains:
  - analyzing blocks and transactions sequentially, considering 625,941 bitcoin blocks (approximately 253.38 GiB) and 6 million ethereum blocks (approximately 107 GiB of blocks and transactions data); and
  - analyzing up to 98 million bitcoin clusters.

Highlighting the problems related to the misuse of steganography in blockchains is essential to the evolution of this promising technology. To effectively address this problem, prevention efforts are needed, but detection efforts are also significant. In this perspective, this work has an additional contribution since it publicizes a detection effort against the use of steganography in blockchains. These detection efforts can discourage malicious users since their actions can now be evidenced and brought to public knowledge due to the analysis. Besides, if the efforts found no evidence of steganography, it means that the blockchain is validated (within the scope of the steganalysis performed). This validation means that the legitimate users can rest assured that, so far, they have not stored data hidden by others. On the other hand, it is worth mentioning that such detection efforts must be continuous and complementary to the prevention efforts (e.g., appropriate content filters and other countermeasures).

### 1.3. Text Organization

This work is structured as follows. Section 2 presents the necessary background on data insertion and hiding in blockchains, discussing the related works. In Section 3, the steganalysis approach is detailed. Section 4 discuss the results of the evaluation in Bitcoin and Ethereum. Section 5 shows the conclusions and lessons learned from this work.

## 2. Background

We divide this section into three parts. The first part (Section 2.1) revises some of the known data insertion techniques for blockchains, focusing on the Bitcoin and Ethereum blockchains. The second part (Section 2.2) summarizes the available steganography techniques proposed for blockchains. In the last part (Section 2.3), we discuss the absence of related work.

### 2.1. Data Insertion in Blockchains

Arbitrary Data insertion in blockchains can be a debatable subject [17,24]. On the one hand, companies provide Proof-of-Existence and notary services in blockchains. For instance, one can store a cryptographic digest of a given electronic document in a blockchain. In this case, the document has proof of its existence guaranteed by the blockchain at a given cost. Such services can be useful for society and have promoted research on blockchain-based notary services [25,26]. Some of the data found in Bitcoin have innocent content, such as a photograph of Nelson Mandela. The most known data inserted is probably the Bitcoin Genesis block’s message (“The Times 03/Jan/2009 Chancellor on the brink of second bailout for banks”) [13,17]. Such content does not impose any serious problems on Bitcoin users.

On the other hand, the work of Matzutt et al. [13] presented an opposing side of the arbitrary data insertion in blockchains. However, other files recovered in Bitcoin are inappropriate or have objectionable content. The so-called full nodes of blockchain networks may have to download all of the data, despite its content, to keep the blockchain’s correctness. Some countries have laws where the mere possession of such content is objectionable. This content is harmful because it discourages the users from participating in the network [27]. Another known negative side of data insertion in blockchains is the performance; it can quickly increase the blockchain’s size, impacting network participants [28].

Table 1 presents the same examples of known data insertion methods in blockchains and blockchain platforms. *On-chain* data insertion is the term used for the data directly inserted in the blockchain. Other platforms allow only *off-chain* data insertion. Off-chain means that only a “link” (such as a hash value) is inserted in the blockchain, requiring external storage for the original data. The examples of platforms given in Table 1 have different characteristics, but most do not allow on-chain arbitrary data insertion.

#### 2.1.1. On-Chain Data

Since its conception, Bitcoin allows the insertions of data on-chain. Specifically, in the Bitcoin blockchain, the available methods for on-chain insertion are listed below [13,17]:

- In Coinbase: the first transaction input of each block in Bitcoin. The Bitcoin miners control it [17]. Coinbase transactions allow the insertion of up to 100 bytes of arbitrary data.
- Using OP_RETURN: since 2014, OP_RETURN is an opcode of Bitcoin which invalidates the transaction. OP_RETURN is the Bitcoin standard of inserting arbitrary data (limited to 83 bytes) in the blockchain, but the outputs are unspendable.
- In Standard transactions: Bitcoin provides five “types” of transactions. They can be misused to insert data in the output scripts. The types are Pay-to-PubkeyHash (P2PKH), Pay-to-Script-Hash (P2SH), the obsolete Pay-to-Pubkey (P2PK), Multi-Signature, and, after 2014, OP_Return. We commonly find P2PK in coinbase transactions from the earlier blocks of the blockchain. The script hash can embed arbitrary data, and miners cannot verify it. An alternative nomenclature is the “Pay-to-Fake-Key”, “Pay-to-Fake-Key-Hash” and “Pay-to-Fake-Multisig” for this type of insertion [17]. Data insertion in standard transactions results in burning coins but allows 57.34 KiB to 96.7 KiB of arbitrary data.
- In Non-standard transactions: such transactions deviate from the standard rules of Bitcoin (e.g., minimum output value, maximum transaction size). It may not be the best way to store data because miners often ignore them, and, if ignored, they will not insert the data into the blockchain. On the other hand, if successful, this type can insert up to 100 KiB of arbitrary data. At the time of writing, this is the current size limit of transactions.

Sward et al. [17] also analyzed the data insertion methods in the Bitcoin blockchain. Their analysis identified other problematic aspects. First, some insertion methods, such as the P2FKH, are computationally inefficient, and the data recovery process can be cumbersome to perform. Secondly, estimates given in their work show that the amount of “burned” BTC coins to store 2.59 MB of ASCII data can reach up to 118.96 BTC coins (3.576.139,83 US Dollars on 27 Jan 2021). This estimate may not be accurate due to their methodology, which considered unspent outputs of P2PKH transactions, and it did not analyze the semantics of the data, where the only criteria were the 18-byte size (at least) and that it must be part of the printable ASCII set. Nevertheless, the estimates suggest a high expense to store a couple of MBs in the Bitcoin blockchain.

#### 2.1.2. Off-Chain Data

The other examples of Table 1 use off-chain data storage. It is also important to analyze such a type due to the replicability of the data between nodes. On-chain data must be present on all of the *full nodes* which participate in the network, and off-chain data is replicated to a set of participant nodes, depending on the blockchain used. Still, some data insertion problems may persist, but with the advantage that we do not contaminate all nodes if there is the insertion of inappropriate data. Besides, the rules for the integrity of off-chain data can be different when comparing to on-chain data. Below, the off-chain examples of Table 1 are discussed.

Starting with the Ethereum example, the data related to Smart contract code (called state) is stored off-chain, and the hash of the data is inserted in the blockchain [29]. This design avoids an uncontrolled increase in the size of the blockchain. Besides, Ethereum provides selfdestruct operation, which is a way to delete a contract, giving a choice for nodes to keep or delete the corresponding storage (data and code). On the other hand, selfdestruct cannot be called arbitrarily, only in specific situations, because it would damage the integrity of legitimate contracts.

The third example is the Hyperledger Fabric, a Blockchain Development Framework [30]. Its focus on deploying permissioned blockchains for enterprise solutions. Regarding data insertion, Hyperledger offers a private *data collection*. The data is stored privately in storing only in authorized organizations’ peers, not in the blockchain but in an external database (called “SideDB”).

NEO is a Blockchain Development Platform, but it provides a public blockchain (called NEO MainNet) for its cryptocurrency [31]. It also provides a file system called NeoFS. The participant nodes are connected and integrated into a distributed file system based on DHT (Distributed Hash Tables) and the blockchain. However, not all nodes can join the network for this function, and it must be registered and pay some security deposit that the node loses if the node starts to misbehave. Most importantly, data is not stored *on-chain*, but, in NeoFS nodes, access to the data happens through smart-contracts. The NEO blockchain has a sidechain for data audit and payment purposes.

The last example in Table 1, Helium blockchain, provides state-channels for increasing the performance of the transactions [32]. Helium focus on giving network access to the participants in the exchange of credits. The State-channels can be viewed as side storage since the corresponding peers have to maintain it. Helium has certain transaction types that include anonymized user data, such as location coordinates and hot spot names, but it is not clear from the documentation if the state-channels can store arbitrary data.

Most of the examples examined in Table 1 shows that on-chain options could present limitations in storage capacity and performance, as well as privacy and cost concerns. To address these on-chain limitations, we use on-chain/off-chain hybrid options, where an external database stores the desired data and, generally, the blockchain stores only the hash of the stored data. Regulations, such as GDPR [33], also impose restrictions on the insertion of personal data in the Blockchain [28]. Regulations can explain why there are not many blockchains allowing random insertion.

### 2.2. Data Hiding in Blockchains

Steganography is part of the information hiding methods used to make data harder to notice [34]. Typically, we used it to hide the fact that a (secret) communication is taking place. The following components are present in a stegosystem: the important or secret message (the *hidden text*) *m*; the unsuspicious document *d*, which is called *covertext* if it contains *m* embedded into; and the *history* *h*, composed by the already transmitted documents in a communication *channel* *C*.

In this work, the authors view the blockchain as the communication channel for steganography. Its primitives are “exploited” in order to hide this communication. The consequence of the substantial integrity of blockchains is that such communication is prevailing, i.e., it will remain stored as long as the network exists. In a stegosystem, we formalize channel *C* as a probability distribution. We view the communication can as a sampling from this distribution. In this sense, the security of a stegosystem relies upon the fact that an observer cannot distinguish the stegotext from objects randomly picked from the channel distribution [35]. This fact applies to the blockchain primitives, such as hashes of addresses and blocks, digital signatures, nonces, and others. The main challenge is to apply the hiding approach in an unsuspicious manner without deviating from the blockchain’s normal functioning.

**Figure 1:**
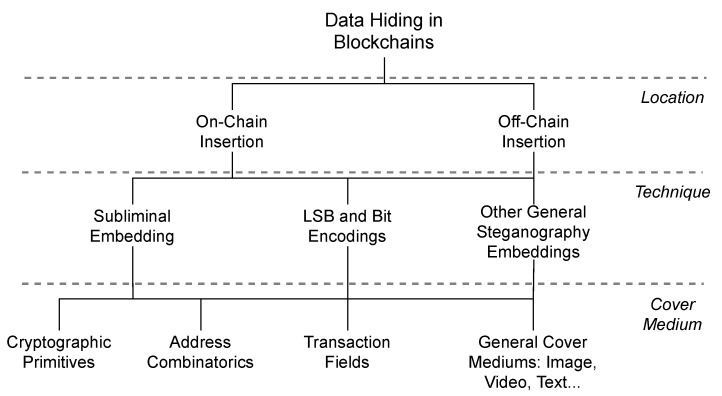
A classification of the blockchain steganography approaches in the literature.

In particular, we classify the state-of-the-art approaches for data hiding in blockchains in Figure 1. The well-known steganography types, such as image-steganography, are included in this classification because they could be present in both on-chain or off-chain data. In this case, one can hide a message in an image and then insert it using the previous data insertion methods (such as OP_RETURN) or storing it off-chain. We can also combine techniques instead of only one, which includes the general methods of steganography. On the other hand, specific approaches for blockchains are the focus of this work. These approaches explore the blockchain primitives for data hiding, for example, transaction addresses and digital signatures. Most of the approaches hide on-chain to the best of our knowledge, and only one work addressed the off-chain data hiding [36].

We present a summary of the specific and on-chain data hiding approaches in Table 2. Regarding the blockchain primitive used as a cover medium, the approaches hide data in transaction fields, cryptographic primitives, or in a permutation of addresses. For instance, we can change the value or a payment address in a transaction to embed secret information. Other approaches exploit the Simmons’ subliminal channel in cryptographic primitives, such as in Digital Signatures [37]. Subliminal embeddings can be viewed as a particular form of steganography since hidden communication is taking place, but the embedding does not alter the cover medium structure [38]. Besides, it is computationally infeasible for an adversary to detect such subliminal messages. Combinations and permutations of addresses in transactions do not fit in Simmons’ definition, but it is classified as a subliminal embedding since it does not change the structure of the blockchain primitive. For completeness, each of the approaches presented in Table 2 is detailed below.

Using the theory behind the Provably Secure Steganography (Hopper et al. [45]), Juha Partala was the first to propose an approach for (provably secure) steganography in blockchains [5]. His approach, called BLOCCE, defines that a payment address is a hash value, and they base the hiding process on the LSB of addresses. Therefore, it hides data in a **Cryptographic Primitive**. First, one encrypts the secret message *m* with a pseudorandom ciphertext function. Then, he orders the addresses so that the LSBs form *m*, including a secret start indicator. The formal specification given by Partala allows the embedding of one bit per block of the blockchain. If there is a pre-computed list of *L* addresses, and if it is significantly greater than *N* (the size of the *m*), then it is easier to sample these addresses in order to match the LSB with the bits of *m*. In theory, Partala’s approach hides information in hashes so that the cover would be classified here as a cryptographic primitive instead of a transaction field. It seems that the research on data hiding in hashes dates back to 2005, with Wu’s “Hash Channels” [46]. However, the detection of this kind of hidden data is still challenging.

In 2018, Frkat et al. [19] proposed a covert communication channel in blockchains called ChainChannels. They designed their approach in the context of botnet control, and it explores the digital signatures in public blockchains for this purpose. Similarly, the approach of Alsalami and Zhang [16,42] exploits subliminal channels in digital signatures but in the perspective of achieving censorship resistance. Their approach embeds information using the random numbers ($c_{j},r_{j}$) of the CryptoNote protocol’s ring signatures. This protocol uses Edwards-curve 25519 with a group order of prime *p* equals 253 bits. The approach uses the least significant 252 bits of $c_{j},r_{j}$. There is a 128-bit IV, where 64-bit is random bits, and the rest are zero to indicate a message’s presence. They use the remaining 376 bits (or 47 bytes) to embed an encrypted *m* with a secret key *k* and the IV. In summary, their approach hides information in a digital signature by using the random numbers of the signing process.

Nine covert channels created with **Address Combinatorics** were proposed by Fionov [18]. His work treats input or output addresses as numbers. A permutation of the addresses is selected to match the hidden data (also represented as a number). This permutation is similar to a rejection sampling process. However, they do not provide enough details on the security and extract the hidden data from these channels. Xu et al. [44] proposed a similar approach, where the blockchain miner performs a so-called *broadcast steganography* (the recipients of the steganographic message are unknown), arranging the payment addresses in a block until it matches the message.

The main drawback of BLOCCE is the low throughput. One can view the approach as a theoretical construction as they did not implement it in practice. To improve BLOCCE, Zhang et al. [39] proposed V-BLOCCE for Bitcoin, where the V is short for Vanitygen, a Bitcoin address generator. It is an approach that hides in **Transaction Fields**, combining the OP_Return data insertion method with Partala’s BLOCCE, where the addresses with hidden information are generated by Vanitygen and indexed in OP_Return transactions. In addition, focusing on Bitcoin, the CCBRSN of Wang and Su [40] was recently proposed. Different from the others, it uses the Regtest network of Bitcoin, which is private. The similarity is that they based their approach in **Transaction Fields**, where they use the transaction addresses to hide indexes of a ciphered version of the secret message. A limitation of CCBRSN relies on a public channel to transmit the ciphered message (defined as a DES-encrypted file).

Interestingly, most of the approaches found so far are either generic or specific for Bitcoin, except for the works of Basuki and Rosiyadi [36], Biryukov et al. [43], and Liu et al. [41]. Basuki and Rosiyadi base their approach on Ethereum smart contracts, hiding data first in images (image-steganography), and then a URL of the image is inserted in **Transaction Fields**. They hide the confidential data outside of the network in their approach, but the URL (and additional parameters) are hidden on-chain, using the LSB with group encodings in Ethereum transactions.

In addition, focusing on the Ethereum blockchain, Liu et al. [41] proposed two covert channels in Ethereum. The authors use the VALUE field in transactions in the following manners: (1) a Multiple-Bit Encoding (MBE) based on HMAC is used to encode a group of bits inside the VALUE field; or (2) a mixed Hash-based MBE scheme, which improves (1) by mixing private data with obfuscation data using hash functions. They base the encoding on two number intervals for hash values previously defined by the communication parties. It can be viewed as a subliminal embedding since the hash of the group of bits of the VALUE field is used to obtain the hidden message. Therefore, the hidden message itself is not stored. The authors argued that Ethereum has advantages over Bitcoin for hiding data, for instance, the faster block confirmation, which ensures that the communication is available (and stored) sooner in the blockchain. However, Ethereum does not allow on-chain arbitrary data in the same way as Bitcoin does.

Biryukov et al. [43] performed a privacy analysis in the ZCash cryptocurrency. ZCash implements a Zero-Knowledge proving system called zk-SNARK [47] to anonymize transactions present in its public blockchain. The authors then present ways to exploit subliminal channels in zk-SNARK cryptographic primitives. Besides, they discussed the impacts of the findings and possible countermeasures.

### 2.3. Related Work

So far, we did not find a specific steganalysis approach for blockchains, except the Frkat thesis [48], which includes the ChainChannels proposal [19] evaluated with pattern analysis. It is worth mentioning that his pattern analysis focused on subliminal embeddings only (in Digital Signatures) and in Bitcoin transactions. The patterns are related to the result of the embedding method and the “lifetime” of transaction outputs [48]. In our previous work, we analyzed Bitcoin to find evidence of steganography use, using statistical analysis. The lessons learned from this case study showed that specific steganalysis approaches are required, mainly due to the following reasons:

1. there already are publications of specific steganography approaches for blockchains, which one can use to embed and hide data (Table 2); and
2. standard steganalysis techniques may not capture specific aspects of these data hiding approaches in blockchains.

Therefore, in this paper, a steganalysis approach is proposed, detailed in the next section.

## 3. A Steganalysis Approach for Blockchains

The primary goal of a steganalysis approach is to retrieve sufficient evidence about the presence of a hidden message [49]. A further goal is a disclosure: to show the meaningful content of the hidden message. Note that successful disclosure may be difficult to achieve if the message is encrypted.

Among the several types of steganalysis, statistical steganalysis aims to determine which statistics provide evidence about an embedding (or hiding) method [50]. The design of a steganalysis approach of this type has two requirements, known as a priori: (i) the characteristics of the cover medium; and, optionally, (ii) how the hiding method works. On the other hand, universal steganalysis is the type in which the detection does not require knowledge about the steganographic technique used [50]. A combination of these types form a universal statistical steganalysis, where the requirement (ii) is not needed, but this combination is not easy to achieve [49].

We describe the proposal of the Steganalysis Approach for Blockchains in this section. We base it on statistical analysis, but it considers the specificities of blockchains. Section 2.2 gave a background about the available hiding methods, the basis for requirement (ii), so it is needed the characteristics of the cover mediums used in blockchains. Besides, the design of this steganalysis approach considers the roles of users of steganography in blockchains.

**Figure 2:**
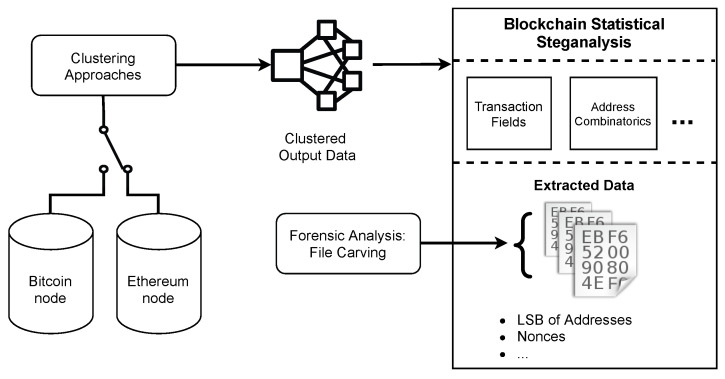
Steganalysis approach proposed for blockchains, instantiated with Bitcoin and Ethereum.

Figure 2 presents the overview of the steganalysis approach proposed. The instantiation uses Bitcoin and Ethereum nodes used in this work, but we can instantiate other blockchains. From the selected node, on-chain or off-chain data is input to clustering approaches. For instance, we can use heuristics to create clusters of transactions in Bitcoin [51]. The clustered output data is then subject to analysis. In this work, transaction fields, such as addresses, values, and nonces, can be analyzed statistically: (i) for each cluster data; or (ii) for a particular cluster of interest. However, we can consider other hiding methods (such as the address combinatorics of Fionov [18]). If the statistics suggest the presence of hidden data, further analysis can disclose the hidden data. From a forensic perspective, File Carving tools can retrieve meaningful data using as input the extracted data (e.g., addresses, nonces, among others).

### 3.1. Statistical Steganalysis

In this approach, steganalysis’s primary purpose is to verify if the data under analysis (e.g., transaction fields) have statistical evidence of steganography. This evidence results from a comparison between the blockchain data with synthetic-generated data (without hidden data). Some hiding methods can leave traces since it changes the structure of the data. However, some subliminal embeddings may not change the cover data structure and, therefore, may not leave statistical traces of modifications, such as a permutation of addresses for hiding data. Therefore, steganalysis has to proceed with the data extraction to check for hidden data or use a different steganalysis type. In addition, the disclosure goal may require decryption techniques (i.e., by brute-force) if the message is encrypted.

The methodology of the statistical analysis is detailed below. First, it depends on the cover medium. In this work, regarding Transaction fields, statistics regarding the Addresses and Nonces are considered, which is similar to the experiments performed in previous work. Secondly, the analysis considers the hiding method employed (such as the LSB). In this work, in order to detect a hidden message embedded with LSB of Addresses, first, a synthetic set of random addresses is generated, and then the following statistics are computed: Shannon’s entropy [52], Average Mean (AM), and the percentage of Monobit test (from FIPS 140-2 [53]) failures. The synthetic dataset statistics are compared to the data under analysis, allowing to detect traces of hidden data. We did not generate a synthetic dataset of nonces, but the same statistics can be applied since we expect that nonces have a random-like behavior. It is worthy of mentioning that other statistics can be computed, depending on the granularity required. Besides, we only considered on-chain data in this work. On the other hand, this approach can also be instantiated for off-chain data, requiring, for instance, a different set of statistics depending on the cover medium (e.g., images).

### 3.2. Clustering Approaches

Including clustering in the blockchain, the steganalysis approach is essential. A steganography user who is willing to communicate using the blockchain may not have access to all of the blockchains’ transaction fields due to blockchains’ decentralized design. In this context, we define two roles for a steganography user: (i) the ordinary user, which has access only to his transactions (inputs, value, and outputs), in general; (ii) the miner, which constructs and submits blocks for validation by the network, which has full access to manipulate block data but only in the parts allowed by the network rules. Clustering addresses of a steganography user (miner or standard user) allow the steganalysis approach to retrieve that particular user’s hidden messages. This retrieval means evidence of steganography is bound to a cluster of users and is tamper-proof due to the integrity properties of the blockchain.

Depending on the blockchain under analysis, there are a set of heuristics employed for clustering. Several works deal with heuristics in blockchains [51,54,55,56]. The heuristics mean that, if successful, they aggregate data of the same user or group. The heuristics applicable for Bitcoin or Ethereum are summarized below:

- Multi-Input: Bitcoin clients’ behavior when a user selects a set of his possession addresses to perform a payment. Generally, we make this selection when the available currency of a user spreads among addresses. Then, he aggregates its inputs until it reaches the amount of currency that he wants to spend or transfer.
- Shadow: typically, Bitcoin clients generate a “shadow” (or change) address to send a payment/transfer change. For instance, an address that holds 2 BTC, if used by the user to send 1.5 BTC, the change (0.5 BTC) is sent to a newly generated address in possession of the user. Shadow addresses are further explored in two ways [56]:
  - Checking Address Format: if transactions that fit in the Shadow heuristics and have the same address format (e.g., P2PKH), it probably means that these transaction addresses belong to the same user.
  - Checking Change Value: a Bitcoin rule states that the sum of inputs of a transaction must be equal to or higher than the sum of outputs. The behavior of some Bitcoin clients is that the change value is the minimum value in the list of outputs of the transaction, as follows: if the sum of outputs minus the value of an output *i* is higher than the sum of inputs minus the minimum value in the inputs, then *i* is a change address.
- Deposit Address: regarding Ethereum, Friedhelm [57] argues that this is the most effective clustering technique. One can deposit to a deposit address. A financial exchange controls this address that forwards assets (or tokens) to another address, i.e., the recipient. The clustering technique builds on the assumption that the creation of deposit addresses is per customer (of the exchange). Under this assumption, multiple addresses related to the same deposit address belong to the same user or entity. Friedhelm argues that it is not easy to identify deposit addresses, and he demonstrates it using a specific token network built on ethereum (not for the cryptocurrency itself).

It is important to mention that the clustering of addresses may not be accurate in all scenarios. It may be an imperfect process depending on heuristics [55]. Besides, this operation in blockchains is computationally expensive in terms of memory requirements. For example, BlockSci minimum requirements are 60 GiB of memory RAM [55] and BitIodine requires 64 GiB [51]. On the other hand, we can combine heuristics for better accuracy, and they form an important tool to analyze blockchain data from the users’ perspective: what, when, and to whom their transactions are intended. Potential users of steganography have access only to their transactions in the blockchain. Therefore, the attempts to discover illegal steganography use in their transactions require such clustering approaches. Besides, the clustering gives evidence about the possession and authorship of the hidden data from a forensic perspective when found.

### 3.3. Forensic Analysis

In general, blockchains can store a large number of transactions. The forensic analysis is included in this approach to support the disclosure of evidence of steganography if found. First, blockchains’ size increases the steganalysis process’s difficulty, requiring then automated process or tools. For instance, at the time of this writing, Bitcoin has near to 328 GiB of data [58], and Ethereum can reach up to 670 GiB when selecting a full node of this network [59].

Regarding the size of the blockchain, in this approach, the analysis can be divided into the following ways:

- By time: useful for reconstructing the timeline related to the steganographic evidence. We divided Bitcoin blocks into 6-month wide chunks in previous work, each chunk being subject to steganalysis. This choice depends on the desired level of granularity of the analysis. The chunks may have variable sizes, depending on the blockchain under analysis.
- Clustering addresses: applying clustering approaches means that the steganographic evidence belongs to the cluster. Clustering addresses can relate the evidence with a user (of steganography, in this case) under the heuristics’ assumptions in the process. For example, they used clustering to discover and subsequent seizure of bitcoins of the Silk Road Case in 2013 [51]. Clustering is the focus of this approach, but they are not mutually exclusive options.

For example, the extracted data in the steganalysis process (extracting LSB of Addresses) are subject to further analysis to disclose the steganographic evidence. Such evidence can be more than a text message hidden in the blockchain. Understand what the possible data types of the evidence are is essential, whether it is a JPG image, a video file, a PDF document, or of another type. For finding text-based evidence, string search can be helpful, but it requires contextual information about the hidden message. One can also use language recognition tools in this case [60,61]. Generically, *File Carving* techniques and tools can assist the analysis to retrieve the steganographic evidence. In this approach, we employed the Scalpel tool using the extracted data as input [22]. In the next section, we discuss the results of the steganalysis approach proposed.

### 3.4. Methodology of this Study

The methodology of the Steganographic Analysis performed in this study is based on an instantiation of Figure 2. The main requisite for our steganalysis is the blockchain data obtained previously from the network. We compared two types of analysis: **sequential analysis**, which requires raw blockchain data, and **clustering analysis**, which requires a clustering method to create the clusters of blockchain addresses. Sequential analysis means that each block or transaction is processed sequentially, without any clustering method. On the other hand, the clustering analysis aims to aggregate address data from the same network users or entities.

Algorithm 1 presents the pseudocode for the Steganographic Analysis performed in this study. It receives as input the blockchain blocks, represented as *B*, and the clustered data represented as *C*. If the clustering analysis is selected, it is assumed that *C* contains the required clustered data. Otherwise, *C* is empty, meaning that sequential analysis will be performed. Both types of analysis aim to retrieve the addresses for each cluster or for each transaction in the given block. After obtaining the list of addresses, the function LSB(address) returns the least significant bit is used, which extracts all bits for further analysis. The sequential analysis also retrieves the nonces from the blocks. The function APPEND(data) stores the extracted data from addresses or from nonces. Lastly, the function Statistical_Analysis(AddrData,nonceData) computes the statistics for the given data. The pseudocode for this function is omitted for simplicity, but the selected statistics are presented in Section 3.1. Algorithm 1 returns both the statistical results and the extracted data (nonces and/or LSB of addresses). The extracted data is given for forensic analysis (Section 3.3).
**Algorithm 1** Steganographic_Analysis(Blocks *B*, Clusters *C*)1:**if**C≠{}**then**2:    **for** c=1,2,…, *C* **do**3:        A←c.addresses4:        **for** addr=1,2,…, *A* **do**5:           lsb←LSB(addr)6:           AddrData.APPEND(lsb)7:        **end for**8:    **end for**9:**else**10:    **for** b=1,2,…, *B* **do**11:        T←b.transactions12:        NonceData.APPEND(b.nonce)13:        **for** t=1,2,…, *T* **do**14:           A←t.addresses15:           **for** addr=1,2,…, *A* **do**16:               lsb←LSB(addr)17:               AddrData.APPEND(lsb)18:           **end for**19:        **end for**20:    **end for**21:**end if**22:Stats←Statistical_Analysis(AddrData, NonceData)23:**return**AddrData, NonceData, Stats

We instantiated our approach in two experiments. The first experiment focuses on the sequential analysis of blockchain data. In previous work, we performed this analysis in Bitcoin, and, here, we compare it to Ethereum. Bitcoin and ethereum are very popular, and most of the proposals presented in Section Table 2 target bitcoin or ethereum. The second experiment focuses on clustering analysis. We select Blocksci API for clustering addresses in bitcoin. On the other hand, it seems that there are not many consolidated approaches for clustering addresses in ethereum. Etherclust [57] would be a choice but in the context of ethereum token networks. We did not consider the token networks in our scenario. Besides, it would turn difficult to compare it with our sequential analysis; therefore, we restrict the clustering analysis for bitcoin. We give more details of the experiment below:

1. **Sequential analysis (no clustering)**
  - Datasets:
    - Bitcoin (previous work): we obtained blockchain data from the official Bitcoin client. In numbers, approximately 253.38 GiB in 625,941 blocks (synchronized at 14 April 2020).
    - Ethereum: obtained from Ethereum mainet. We analyzed 6 million blocks of data, which corresponds to 49.62% of ethereum blocks (at 22 March 2021) and approximately 107 GiB (blocks and transactions).
  - Tools: Ethereum ETL [62] parser and Bitcoin parser [63] to extract and parse blockchain data.
  - Scope: Statistical analysis of the LSB of addresses in transactions and Nonces of block headers. We considered only on-chain data. Scope includes the forensic analysis over the extracted data, aided by Scalpel.
2. **Clustering analysis:**
  - Cluster sets: Two bitcoin cluster sets, considering the first 3.3 GiB and 28 GiB, respectively, of blockchain data.
  - Tools: Blocksci API [55] for parsing and clustering bitcoin data;
    and Scalpel tool [22] for file carving in the extracted data.
  - Clustering methods (Section 3.2): we selected the multi-input heuristic of Blocksci.
  - Scope: similar to the sequential analysis. The difference is that we clustered the addresses; therefore, only the LSB of addresses were investigated.

Our implementation is publicly available [64], designed to automate parts of the analysis presented in Figure 2. Depending on the configuration, it performs blockchain parsing followed by sequential analysis (e.g., statistics of LSB of addresses); or adds a clustering method before the analysis. Our implementation computes the statistics with the extracted transaction fields from the dataset or each cluster. The extracted data includes LSB of addresses and nonce data: Least-Significant Byte (LSByte) and Most-Significant Byte (MSByte) of Nonces. In the second experiment, we restricted the scope to the output addresses (bitcoin only). Etherclust [57] could be used for ethereum, but it restricts the analysis to tokens of the ethereum network; thereby, we focused on bitcoin in the second experiment. We further analyzed the extracted data with file carving (separately from our implementation). For the carving, we enabled all of the magic numbers available at Scalpel. We describe the results of both experiments in the next section.

## 4. Results

We present the sequential analysis first, considering block nonces (Section 4.1.1) and LSB of addresses (Section 4.1.2) of bitcoin and ethereum blockchains. We discuss the results of the clustering analysis in Section 4.2. Each analysis used a different dataset of extracted blocks from the blockchain. Table 3 presents the overview of the datasets investigated in each experiment, including the division of blockchain data into chunks and the clustering results. We divide the bitcoin into 23 variable-size data chunks by their timestamp (for every six months). This time division resulted in variable sizes from 3.95 MiB up to 26.30 GiB. In the case of ethereum, we divide the blockchain differently. Ethereum-etl parser returns filtered data separately (blocks, transactions, token transfers); therefore, we decided to work with chunks of fixed size. This division resulted in 119 chunks of nonces (500.000 nonces/chunk) and 2720 chunks of addresses (1 M addresses/chunk).

Regarding the clustering results, we generated two cluster sets with Blocksci: ~5.76 million of clusters from the first 3.3 GiB; and 98.26 million of clusters from the first 28 GiB of the bitcoin blockchain. Blocksci API allows driving the analysis by address type: pubkey, P2PKH, and P2SH. In this case, we extracted address data by type in each cluster. Supplementarily, we also give a compilation of the statistics of both analyses in our GitHub repository.

### 4.1. Sequential Analysis

Previous work found no evidence after a sequential analysis in Bitcoin, although it shows some interesting results. Here, we extend this analysis to Ethereum and further compare the results between the cryptocurrencies. The nonces in ethereum are the block nonces (same as in bitcoin), and we discarded transaction nonces. First, we analyze the data inside of nonces, which blockchain miners manipulate. Then, we analyze the LSB of output addresses in transactions.

#### 4.1.1. Block Nonces

**Figure 3:**
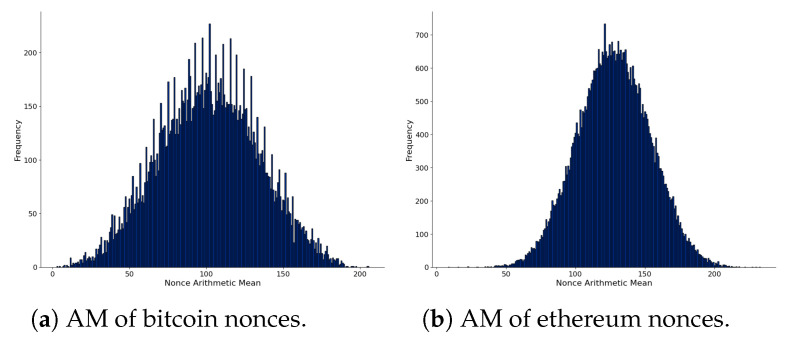
Comparison of nonces of bitcoin (**left**) and ethereum (**right**) from the first chunk of parsed blockchain data. AM is the Arithmetic Mean of each nonce.

We present the AM distribution of nonces considering the first chunk of data in Figure 3. The left part shows the AM distribution computed from 32-bit nonces of bitcoin, and the right part comes from 64-bit nonces of ethereum blocks. The distributions are different: bitcoin is not close to random, but ethereum is much more close. This fact is interesting since both cryptocurrencies perform a similar proof-of-work consensus mechanism, which uses the nonces as part of their process (at this time of writing). Although there are different implementations available, reports indicate that miners of both cryptocurrencies compute nonces incrementally [65,66].

**Figure 4:**
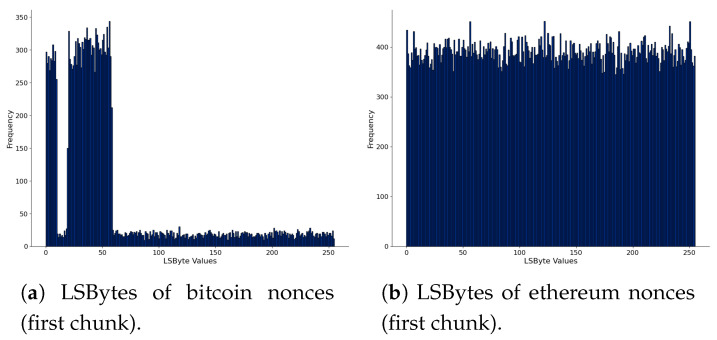

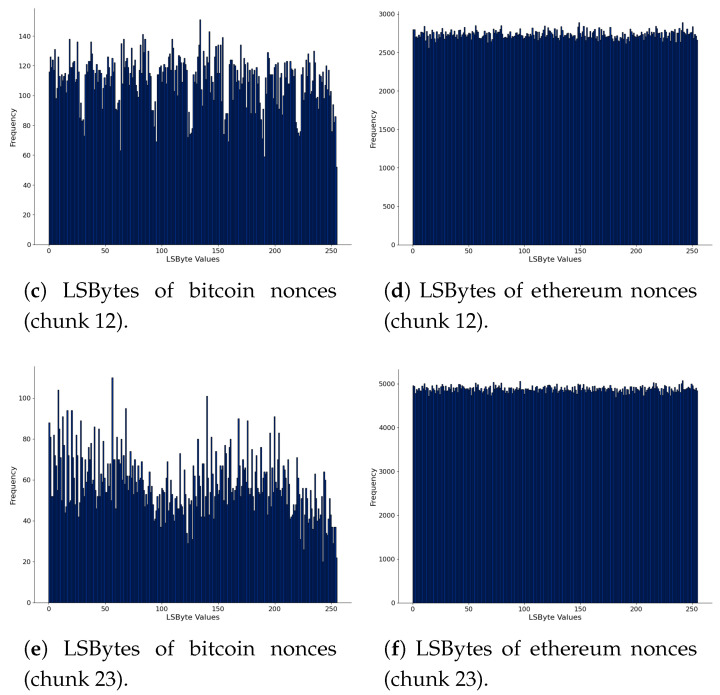
Comparison of LSBytes of nonces of bitcoin (**left**) and ethereum (**right**), considering the first and the last chunk of parsed blockchain data.

We further analyze the nonces in Figure 4, where we present the Least Significant Bytes (“LSByte”) of nonces. Figure 4a,c,e are of bitcoin, and Figure 4b,d,f are for ethereum. We computed all data chunks’ histograms to see the variations, but we present three: the first, chunk 12, and chunk 22, arbitrarily chosen. In bitcoin, there are different patterns in the histograms. One hypothesis is that these patterns observed in nonces could indicate hidden messages or arbitrary data. In ethereum, nonce values are more uniformly distributed, and this behavior has not changed among the blockchain data chunks. However, the bitcoin mining pools and the use of the extra nonce field in bitcoin blocks can be an explanation of the observed patterns in the histograms. Therefore, there is no concrete evidence that the nonces carry hidden messages using this sequential analysis.

#### 4.1.2. LSB of Addresses

In this experiment, we analyze the LSB of addresses by comparing the extracted data statistics with a dataset with LSB of synthetic-generated hashes. The synthetic dataset statistics would be the expected result if the addresses were generated correctly, i.e., without modifications. Bitcoin uses SHA256, and Ethereum uses Keccak SHA-3. If the statistics are different from the expected, it could indicate manipulation on the LSBs.

Table 4 presents the results of the LSB statistics in this sequential analysis. All of the chunks were analyzed, but here we present chunk 14, arbitrarily chosen. For bitcoin, the addresses of transactions are grouped by type: *coinbase*, *pubkey*, *Pay-to-Public-key-Hash* (P2PKH) and *Pay-to-Script-Hash* (P2SH). The *coinbase* here is the address (or addresses) in the output of each block’s first transaction. In the case of ethereum, we exported addresses without distinction, but we discard contract creation addresses. The focus is on Externally Owned Accounts (EOA) addresses.

The results presented in Table 4 suggest that some LSB statistics are far from the expected (comparing with the synthetic dataset). For example, bitcoin *pubkey* extracted LSBs look far from random. We can explain such results due to the repetition of addresses found in transactions, which is a problem with the sequential analysis since an address can be reused in transactions, although not recommended in bitcoin. Besides, this behavior can be seen in other chunks, as well.

Regarding ethereum, the overall behavior of LSB addresses does not indicate the presence of messages. For instance, the overall monobit test failure rate is 1.47% of all chunks. On the other hand, chunk 14 fails in monobit, and a granular analysis is needed (i.e., for each chunk). In addition, the reuse of addresses happens since it implements the accountable model. Chunk 14 has a sequence of 12 bytes with value 0xFF, which indicates that those 96 bits (with value = 1) come from the same address. This fact is the main problem of sequential analysis. Still, for completeness, all chunks were further analyzed by scalpel, and we present the results in Section 4.3.

### 4.2. Clustering Analysis

We compute the statistics of the LSB of addresses that belong to a cluster. Each cluster is analyzed separately, which simulates the users’ perspective, but limits the chosen clustering method’s assumptions. For instance, a bitcoin user controls only his addresses, and, if he hid a message using LSBs, one could scatter this message among different transactions and blocks. Therefore, we first computed each cluster, and for each cluster, we extract the LSB data for analysis.

We present a summary of the results in Table 5. It presents the clusterer with the input blockchain data used, the maximum and average size of LSB data found in clusters. Besides, we select the most significant cluster to present its statistics.

From the results of Table 5, it is clear that clusters in Bitcoin could carry steganography data up to 360 KiB if used for such a purpose. On the other hand, on average, this value is lower (less than 1 KiB). This result builds on the assumption that the clustering methods are accurate. If not, the extracted data is an aggregation of addresses from different entities, not from a single user. Besides, our cluster analysis did not encompass all of the blockchain data, which means that cluster sizes can be more extensive. Therefore, they would be capable of carrying more steganography data than we have found in our analysis. In addition, we only found pubkey and P2PKH types, probably due to the reduced blockchain data (at most 28 GiB). We found no P2SH data.

It is infeasible to present here the statistics of all clusters. For example, Blocksci (with 28 GiB of bitcoin blockchain) generates 98 million clusters. Therefore, we selected the cluster with more addresses to present its statistics in Table 5. In summary, all of the analyzed clusters and statistics did not suggest the presence of hidden messages. For instance, we did not find monobit failures in Blocksci clusters. This result is different from the sequential analysis, where monobit failures were found. The statistics of the clustering analysis indicate that there was no address manipulation in the clusters. However, if encryptions are used in hidden messages before the LSB embedding, we cannot detect them using our statistical analysis.

### 4.3. File Carving Results

We further analyze the extracted data of each analysis (sequential and clustering) using scalpel tool. Almost all of the magic numbers of scalpel were enabled, but, after the bitcoin analysis, we disabled some of them to avoid a high number of false positives. We provide the configuration of scalpel used for reproducibility. Nevertheless, we found only false positives in the extracted LSB data. They are presented in Table 6.

The results of the sequential analysis show that we extracted more LSB data from bitcoin than from ethereum. The input dataset was more significant for bitcoin, but it is worthy to note that we excluded smart-contract addresses; we only considered EOA addresses. At the time of writing, we compute contract addresses in ethereum by hashing the creator’s address concatenated with the number of transactions that the creator has sent. We believe that matching steganographic messages in such addresses is more complicated.

The total data extracted from Blocksci (with an input of 28 GiB) is approximately 1.45 MiB, summing up the LSB data from all clusters. Only small files could be embedded in the blockchain in the clustering analysis, limited to the larger cluster found. However, scalpel in the extracted LSB data has found no file, which means that we have not found any steganography evidence in the LSB of addresses. On the other hand, we only analyzed part of the blockchain, and we built our results on the assumption of the clustering methods.

## 5. Conclusions

In this work, we proposed a steganalysis approach for blockchains and evaluated it in Bitcoin and Ethereum. We compared the sequential and clustering types of analysis. We found out that clustering addresses allow a better analysis compared to sequential analysis since it aggregates the data that belongs to an entity or user of the blockchain network. This means that this approach helps to identify if a steganography user hid data in his cluster of addresses. In both experiments, the overall results support that there is no concrete evidence of steganographic messages, considering the scope of our steganalysis proposal.

In this study, we also proposed a classification of blockchain steganography approaches as the basis for our steganalysis. This classification would help researchers and practitioners to have a summarized view of the available techniques focused on blockchains. Understanding how attackers could hide data in blockchains is the first step towards the protection against such threats. For reproducibility, we provided the implementation for our steganalysis proposal, which is built to find evidence of steganographic messages in bitcoin and ethereum blockchains.

In the first experiment, we sequentially analyze the blockchains, block by block. Due to the size of the blockchains, it is safe to conclude that this size is one of the difficulties in detecting steganography. Statistics from block nonces and addresses were computed to find traces of modifications in transaction fields. This analysis is simpler to perform, but it does not capture the perspective of blockchain users. Users’ transactions may not be inserted sequentially in the blockchain; therefore, it acts as an additional secrecy level for this kind of steganography. Therefore, we employed clustering methods in order to analyze such a scenario. Additionally, we carved the data from the sequential analysis, and we found no evidence of steganography in this experiment.

An interesting finding is showed by the clustering analysis. Bitcoin multi-input clusters could carry up to 360 KiB of LSB steganography data. This means that a bitcoin user could have 360 KiB of arbitrary data hidden in his addresses. This result could lead to different applications, where the (hidden) data possess high availability because the blocks are spread across the network, and the user has a level of anonymity provided by the blockchain. In the perspective of a malicious user, he could explore this space in the blockchain for bad purposes, e.g., to communicate a secret attack plan (or something illegal). On the other hand, based on the results of our experiments, our steganalysis did not show any evidence of hidden messages, and the forensic analysis applied in the clusters showed no trace of meaningful content.

We cannot conclude that bitcoin and ethereum are completely free of steganographic messages. We analyzed a significant part of the blockchains, but the completeness requires a continuous effort since the blockchain grows every day. Regarding the clustering analysis, users could employ a clustering evasion technique that can obfuscate the analysis. In addition, we highlight that our results are based on the accuracy of the clustering methods and the scope of the steganalysis.

Unfortunately, the scope of our analysis left out the following aspects, which are considered for future work:

- There are many blockchains available, with different characteristics, which can also be analyzed for steganography use.
- To the best of our knowledge, no work discussed the impacts of off-chain data hiding approaches in steganalysis. Although this topic mixes with standard forms of steganography (e.g., image-steganography), it may add difficulties in detection since the data can be sparse in different network locations. For instance, some blockchains provide private off-chain storage through the network, imposing a problem from a steganalysis perspective.
- The effectiveness of our clustering analysis is dependent on the success of the clustering methods. Not all methods were evaluated in our work. It is important to note that cluster size and clustering evasion (e.g., mixing approaches) directly impact the steganalysis’s effectiveness and time.

Providing specific steganalysis approaches for the blockchains is essential. It can assist the protection against steganography in blockchains. However, we recommend that the protection efforts should be employed continuously. The main reason is that zero-day steganography techniques could exploit blockchains without detection or countermeasure. In this sense, we conclude that more research in steganalysis for blockchains is required. Such research effort would contribute to the security and the adoption of this promising technology.

## Figures and Tables

**Table 1:** Examples of data insertion methods in popular blockchains.

Example	Type/Application	Public?	Data Insertion Methods
Bitcoin	Cryptocurrency	Yes	Coinbase, OP_Return, Standard and Non-Standard transactions
Ethereum	Cryptocurrency, Smart Contracts	Yes	Smart Contract state stored in a key-value like database (off-chain), and the hash is stored on-chain.
HyperLedger	Blockchain Platform	No	Private data collection: inserts arbitrary data off-chain
NEO	Cryptocurrency, Blockchain Platform	Yes	NeoFS stores data off-chain only smart contract code is stored on-chain
Helium	Cryptocurrency IoT & Wireless Networks	Yes	State-Channels for off-chain data (also allows transactions)

**Table 2:** Summary of data hiding approaches for blockchains accordingly to the cover medium.

	Transaction Fields	Cryptographic Primitives	Address Combinatorics
Data Hiding Approach	Basuki and Rosiyadi, 2019 [36] Zhang et al., 2020 [39] Wang and Su, 2020 [40] Liu et al., 2020 [41]	Partala, 2018. [5] Frkat et al., 2018 [19] Alsalami and Zhang, 2018–2019 [16,42] Biryukov et al., 2019 [43]	Fionov, 2019 [18] Xu et al., 2019 [44]

**Table 3:** Summary of the datasets manipulated in each experiment.

Blockchain	Experiment Analysis	Dataset	Chunk/Cluster Size	Quantity of Chunks/Clusters
Bitcoin	Sequential	253.38 GiB, 625,941 blocks	Variable	23 chunks
	Clustering	Blocksci: 3.33 GiB	Variable	5763155 clusters
	Clustering	Blocksci: 28 GiB	Variable	98266113 clusters
Ethereum	Sequential	~107 GiB, 6 million blocks	0.5 M nonces, 1 M addresses	119 chunks (nonces), 2720 (addresses)

**Table 4:** Results of the statistical analysis of the chunk number 14.

Address Data	Dataset	Entropy	Arithmetic Mean (AM)	Monobit Failures
(Synthetic) SHA256 LSB	1 Gbit	7.999998	127.5034	~0.012%
*coinbase* LSB	14.2 KiB	7.966716	119.6772	100.00%
*pubkey* LSB	10.2 KiB KiB	1.392768	98.0966	100%
P2PKH LSB	10.5 MiB	7.982699	128.2420	27.87%
P2SH LSB	625.8 KiB	7.899532	137.4060	94.80%
(Synthetic) Keccak-256 LSB	1 Gbit	7.999999	127.5016	~0.068%
Ethereum EOA LSB	12.5 KiB	7.472802	107.0722	100%

**Table 5:** Summary of the results of the clustering analysis.

Clusterer	Average Cluster LSB Data	Max. LSB Data in a Cluster	Statistics
Blocksci LSB (1) (3.33 GiB)	721.56 bytes	26 KiB ($C_{tag}$: $2330326_{P2PKH}$)	Entropy: 7.993215 AM: 127.3905 Monobit${}_{Failures}$: 0%
Blocksci LSB (2) (28 GiB)	584.92 bytes	360 KiB ($C_{tag}$: $221_{P2PKH}$)	Entropy: 7.999426 AM: 127.4816 Monobit${}_{Failures}$: 0%

**Table 6:** Summary of the file carving results by analysis type, considering the extracted LSB of addresses.

Analysis Type	Total Extracted Size (Approx.)	Scalpel Output (Files)	False Positives?
Sequential (bitcoin)	161.54 MiB	RPM, PGP	All
Sequential (ethereum)	34.52 MiB	FWS, MPG, PGP	All
Clustering (blocksci—28 GiB)	1.45 MiB	None	N/A
Clustering (blocksci—3.33 GiB)	288.9 KiB	None	N/A

## Data Availability

Not applicable.
